# Reorganization of 3D genome architecture across wild boar and Bama pig adipose tissues

**DOI:** 10.1186/s40104-022-00679-2

**Published:** 2022-03-12

**Authors:** Jiaman Zhang, Pengliang Liu, Mengnan He, Yujie Wang, Hua Kui, Long Jin, Diyan Li, Mingzhou Li

**Affiliations:** Institute of Animal Genetics and Breeding, College of Animal Science and Technology, Sichuan Agricultural University, Chengdu, 611130 China

**Keywords:** 3D genome organization, A/B compartments, Adipose tissues, PEI, Pig breeds, TAD

## Abstract

**Background:**

A growing body of evidence has revealed that the mammalian genome is organized into hierarchical layers that are closely correlated with and may even be causally linked with variations in gene expression. Recent studies have characterized chromatin organization in various porcine tissues and cell types and compared them among species and during the early development of pigs. However, how chromatin organization differs among pig breeds is poorly understood.

**Results:**

In this study, we investigated the 3D genome organization and performed transcriptome characterization of two adipose depots (upper layer of backfat [ULB] and greater omentum [GOM]) in wild boars and Bama pigs; the latter is a typical indigenous pig in China. We found that over 95% of the A/B compartments and topologically associating domains (TADs) are stable between wild boars and Bama pigs. In contrast, more than 70% of promoter-enhancer interactions (PEIs) are dynamic and widespread, involving over a thousand genes. Alterations in chromatin structure are associated with changes in the expression of genes that are involved in widespread biological functions such as basic cellular functions, endocrine function, energy metabolism and the immune response. Approximately 95% and 97% of the genes associated with reorganized A/B compartments and PEIs in the two pig breeds differed between GOM and ULB, respectively.

**Conclusions:**

We reported 3D genome organization in adipose depots from different pig breeds. In a comparison of Bama pigs and wild boar, large-scale compartments and TADs were mostly conserved, while fine-scale PEIs were extensively reorganized. The chromatin architecture in these two pig breeds was reorganized in an adipose depot-specific manner. These results contribute to determining the regulatory mechanism of phenotypic differences between Bama pigs and wild boar.

**Supplementary Information:**

The online version contains supplementary material available at 10.1186/s40104-022-00679-2.

## Background

Studies of 3D genome organization have suggested that the mammalian genome is organized into hierarchical layers [1, 2]. Several levels of structure are known to be important for intra-chromosomal organization: A/B compartments, topologically associating domains (TADs) and promoter-enhancer interactions (PEIs). A genome-wide chromosome conformation capture (Hi-C) study showed that chromosomes segregate into two chromosome compartments termed the A compartment and the B compartment [3]. The genomic regions in the A compartment generally contain transcribed genes and genes with active histone modifications [3] and are thus considered ‘active’ regions. Conversely, regions in the B compartment tend to contain inactivated genes that bear histone modifications associated with transcriptional repression [3], thus representing ‘inactive’ organization. On a fine scale, chromosomes can be divided into spatially insulated genomic regions that are referred to as TADs [4]. These self-interacting TADs, which range from 0.2 to 1.0 Mb in size, are considered structural and functional units of chromosomes [5, 6]. At high resolution, usually more than 10 kb, contacts that directly connect regulatory elements (such as interactions between promoters and enhancers) can be detected [4].

A growing body of studies suggests that the 3D architecture of the mammalian genome plays an essential role in controlling gene transcription [7–9]. While studies of nuclear architecture are advanced in humans and in a few model organisms such as mice, studies of agriculturally important livestock species, including pigs, are currently at a preliminary stage. In 2019, the FAANG consortium published initial 3D genome structures for pigs and also provided 3D genome maps for three other livestock species (cattle, goats, and chickens); these structures revealed a general conservation of TAD boundaries and A/B compartment states among these livestock species [10]. A recent study also revealed analogous evolutionary conservation of TAD boundaries between pigs and humans [11]. In addition, current Hi-C studies in pigs have explored chromatin changes that occur during pig development. For instance, two recent studies found dynamic changes in chromatin structure during pig muscle development in muscle tissue [12] and at the cellular level [13]. However, the variations in chromatin structure among different pig breeds are largely unknown and are important for fundamental studies on pigs.

Here, we generated Hi-C data for two adipose tissue (AT) depots from Bama pigs and wild boars. The Bama pig is a typical indigenous breed in South China and has been considered a fat-type pig breed in several studies [14, 15]. In contrast, wild boars have a high lean meat rate and low fat content, representing the lean type [16]. Through comparative analyses of wild boar and Bama pig, we identified breed-specific chromatin structures in the A/B compartment, TAD and PEI levels. These structural reorganizations were associated with changes in gene expression, and genes specifically detected in wild boar- and Bama pig-specific chromatin structures showed distinct functional characteristics. Our results reveal how chromatin structure differs in two pig breeds.

## Materials and methods

### Ethics statement

All animals used in this study were handled in accordance with the guidelines of the Administration of Affairs Concerning Experimental Animals established by the Ministry of Science and Technology of China, and the experimental procedures used in the study were approved by the Institutional Animal Care and Use Committee in College of Animal Science and Technology, Sichuan Agricultural University, Sichuan, China under permit No. DKY-2019202005.

### Animals and sample collection

In this study, the upper layer of backfat (ULB) and the greater omentum (GOM) were collected from four healthy 2-year-old female pigs (two wild boars and two Bama pigs). The wild boars were brought from the wild to captivity at an early age, and the Bama pigs belonged to a normal Bama Xiang pig line that had not been inbred for a long time. The wild boars and Bama pigs were raised on an experimental farm at Sichuan Agricultural University in Ya’an, Sichuan, China. All pigs were raised in single pens, they were allowed to exercise and were fed under the same feeding conditions. They were fed twice daily and had ad libitum access to water. The animals’ diet was formulated to meet the nutrient requirements recommended by the National Research Council (2012) and the Chinese National Feeding Standard for lean-fat type pigs (NY/T 65–2004) (Additional file 1: Table S1). The pigs were humanely sacrificed after 12 h of fasting in accordance with national regulations for the care and use of animals in research. All samples were immediately homogenized in liquid nitrogen and stored at − 80 °C until use in Hi-C and RNA-seq library preparation.

### In situ Hi-C library preparation

We separately constructed four Hi-C libraries for wild boar ATs (two libraries for ULB and GOM, separately) and three libraries for Bama pig ATs (one library for ULB and two libraries for GOM) according to a previously described in situ Hi-C method with some modifications [2]. We also downloaded one corresponding Hi-C library for ULB in Bama pig, which was generated using the same experimental protocol, from our previous study [17]. Briefly, 1 g of adipose tissue was cross-linked in 4% freshly prepared formaldehyde (Sigma Aldrich, Louis, MO, USA) for 30 min at room temperature, followed by quenching with glycine (Amresco, Solon, OH, USA) at a final concentration of 0.25 mol/L. The mixture was then centrifuged at 1500 × *g* for 10 min at room temperature. The upper layer containing adipocytes were added to 1 mL lysis buffer (9.1 μL 1 mol/L Tris-HCl [pH 8.0] [Invitrogen, Carlsbad, CA, USA], 9.1 μL 1 mol/L NaCl, 18.2 μL 10% CA-630 [Sigma Aldrich, Louis, MO, USA], 50 μL protease inhibitors [Sigma Aldrich, Louis, MO, USA], and 913.6 μL nuclease-free water [Ambion, Carlsbad, CA, USA]) and homogenized with a Dunce homogenizer. The homogenate was centrifuged at 5000 × *g* to collect cell nuclei. The pellet of nuclei was washed twice with 500 μL 1× NEBuffer 2 (NEB, Ipswich, MA, USA) followed by centrifugation at 5000 r/min for 5 min. The pellet was resuspended in 100 μL 1× NEBuffer 2, and SDS (Amresco, Solon, OH, USA) was added to a final concentration of 0.1%. The mixture was incubated for 10 min at 65 °C; Triton X-100 (Sigma Aldrich, Louis, MO, USA) was then added to a final concentration of 1%, and incubated for 15 min at 37 °C. Nuclei were permeabilized, and DNA was digested with 200 units MboI (NEB, Ipswich, MA, USA) at 37 °C for 1 h, 65 °C for 20 min and 25 °C for 5 min. Then, 25 μL fill-in master mix (7.5 nmol/L each biotin-14-dATP, dCTP, dGTP, and dTTP, 25 U Klenow fragment [NEB, Ipswich, MA, USA]) was added, and the mixture was incubated at 37 °C for 45 min to fill restriction fragments and add biotin labels. After inactivation of the enzyme at 75 °C for 20 min, we performed DNA fragment ligation by adding 163 μL of ligation mix (157 μL 2 × Rapid Ligation Buffer and 6 μL T4 DNA ligase [Enzymatics, Beverly, MA, USA]) and incubating the sample at 20 °C for 30 min. The mixture was centrifuged for 5 min at 5000 r/min. After removal of the supernatant, the pellet was resuspended in 20 μL 10 × T4 DNA ligase buffer and 90 μL nuclease-free water, and 50 μL Proteinase K (20 mg/mL) (Tiangen, Beijing, China) and 20 μL SDS were added. The mixture was incubated at 55 °C for 30 min to digest proteins; 20 μL of 5 mol/L NaCl was then added, and the mixture was incubated sequentially at 65 °C for 90 min and at 25 °C for 5 min. To purify DNA, 0.8 × AMPure XP Beads (Beckman Coulter, Brea, CA, USA) were added, and the sample was incubated at room temperature for 5 min. After two washes in 80% ethanol (30 s each time, room temperature) and drying for 3 min, the beads were incubated in 70 μL elution buffer at room temperature for 5 min. To remove nonligated biotinylated DNA, we mixed 2 μg DNA with 10 μL 10 × NEBuffer 2, 1 μL 10 mmol/L dATP, 1 μL 10 mmol/L dGTP, 1 μL 10 mg/mL bovine serum albumin (Sigma, Louis, MO, USA), 1 μL T4 DNA polymerase (Enzymatics, Beverly, MA, USA) and nuclease-free water in a total volume of 100 μL. The mixture was incubated at 12 °C for 2 h. The DNA was then sonicated into 300–500 bp fragments using a Covaris S220 sonicator. The DNA fragments were incubated with 1 × M280 beads (Invitrogen, Carlsbad, CA, USA) at 20 °C for 20 min, washed twice with 200 μL Washing Buffer I (100 μL binding buffer, 99.9 μL nuclease-free water and 0.1 μL Tween 20 [Sigma Aldrich, Louis, MO, USA]) and twice with 200 μL buffer EB (Qiagen, Valencia, CA, USA). We then performed end repair, A-tailing, adapter ligation, postligation cleanup and PCR amplification (8–10 cycles) according to the directions provided with the KAPA Hyper Prep Kit (Roche, Pleasanton, CA, USA). Amplified fragments between 300 and 800 bp in size were then isolated using AMPure XP Beads, and the libraries were sequenced on an Illumina NovaSeq 6000 sequencer (paired-end sequencing with 150 bp read length).

### Hi-C data preprocessing and normalization

Hi-C data were processed using the Juicer pipeline (version 1.5.6) as described in a previous report [18]. Briefly, read pairs were aligned against the reference genome of pig (*Sscrofa* 11.1) using BWA (version 0.7.8) [19] with default parameters. Invalid read pairs, including duplications, low-quality alignment read pairs (MAPQ < 30) and intrafragment read pairs, were filtered out. For merged samples, valid read pairs from each biological replicate were filtered out using similar steps. Information on the *cis*/*trans* interaction ratio and interaction distance was also output by the Juicer pipeline.

The raw intra-chromosomal observed contact matrices were generated using valid read pairs at different resolutions (500, 100, 20 and 10 Kb), and the matrices were then normalized using the Knight-Ruiz (KR) algorithm (removing intrinsic biases within the matrix) [18] as implemented in the Juicer pipeline with default parameters followed by the quantile algorithm (removing biases between matrices) as implemented in the R package “bnbc” (version 1.0.0) with default parameters [20].

Distance-dependent decay of *cis* contact frequencies was analyzed by calculating the median contact frequency per given genomic distance separated by 100 kb of each chromosome based on KR normalized intra-chromosomal observed contact matrices. The mean contact frequencies of all chromosomes at various genomic distances are shown.

The correlation between normalized matrices was calculated using HiCRep [21] by the R package “hicrep” (version 1.10.0) with default parameters based on KR and quantile normalized intra-chromosomal observed contact matrices at 100-kb resolution.

### Generation of inter-chromosomal observed/expected contact matrices

The raw inter-chromosomal contact matrices were generated using valid read pairs at 500-kb, and the matrices were then normalized using the KR algorithm as implemented in the Juicer pipeline with default parameters. To obtain the expected number of inter-chromosomal contacts for each chromosome pair *i, j*, we multiplied the fraction of inter-chromosomal reads containing *i* by the fraction of inter-chromosomal reads containing *j* and then multiplied the result by the total number of inter-chromosomal reads. We finally computed the inter-chromosomal observed/expected contact matrices by dividing the number of actual observed contacts between *i* and *j* by the expected value.

### Chromatin 3D modeling

We used the Python package miniMDS (https://github.com/seqcode/miniMDS) [22] with default parameters, an approximation of the multidimensional scaling (MDS) method, to infer the 3D chromosome conformations based on the KR normalized intra-chromosomal observed contact matrices at 100-kb resolution and the KR normalized inter-chromosomal observed contact matrices at 500-kb resolution. PyMOL (version 2.5.2) was used to simulate the 3D organizational structure of the genome.

### Identification of compartments A and B

Compartments A and B were defined at 20, 100 and 500 Kb resolution by generating PC1 vectors as described in a previous report [3]. Briefly, we first used KR and quantile normalized intra-chromosomal observed contact matrices to generate observed/expected contact matrices. For the loci *i, j*, we calculated the median observed contact frequency at the same distance on the chromosome as the expected contact frequency. The observed/expected contact matrices were computed by dividing the observed contact frequency by the expected contact frequency for each pair of loci *i, j*. We then used the ‘prcomp’ function (default parameters) in R (version 3.6.1) on the observed/expected contact matrices to generate PC1 vectors. We calculated the number of transcriptional start sites (TSSs) in the region 100 kb upstream to 100 kb downstream of each bin as the gene density of each bin. Compartments A and B were determined using Pearson’s correlation between PC1 and the gene density of each chromosome calculated by the ‘cor.test’ function in R (version 3.6.1). If the chromosome showed a positive Pearson’s correlation coefficient, the bins with positive PC1 values in this chromosome were assigned to compartment A, and the bins with negative PC1 values were assigned to compartment B. In contrast, if the chromosome showed a negative Pearson’s correlation coefficient, the bins with positive PC1 values in this chromosome were assigned to compartment B, and the bins with negative PC1 values were assigned to compartment A. We considered regions in which all pairs of replicates showed differences in compartment status between wild boar and Bama pigs to be AB switching regions and genes whose TSSs were located within these regions to be AB switching genes.

To quantify the A/B state of local genomic regions, we calculated the A-B index (representing the likelihood of a sequence interacting with the A or B compartment) at 20-kb resolution as described in a previous report [23] with minor modifications. Briefly, for each 20-kb bin, we calculated the median of the distance-normalized contact frequency (i.e., the observed/expected contact frequency) between it and all A compartment regions on its own chromosome at 20-kb resolution as the A score. In contrast, the median of the distance-normalized contact frequency between this 20-kb bin and all B compartment regions was calculated as the B score. The annotation information on the A/B compartment was obtained from the previously calculated 100-kb PC1. The AB index was then obtained by subtracting the B score from the A score. The higher the AB index is, the more inclined this region is to the A compartment state.

### Identification of topologically associated domain (TAD) and breed-specific TAD boundaries

We identified TADs by combining a previously reported directionality index (DI) [5] and an insulation score (IS) [24] method based on KR and quantile normalized intra-chromosomal observed contact matrices at 20-kb resolution. In brief, we first identified TADs by DI algorithm [5] (hereafter referred to as DI-TAD). Specifically, the DI, a statistical value that reflects the degree of bias between the upstream and downstream interactions of a given bin, was calculated from 2 Mb upstream to 2 Mb downstream along the center of each bin at 20-kb resolution as implemented in the original public code of the DomainCaller [5] with default parameters (http://bioinformatics-renlab.ucsd.edu/collaborations/sid/domaincall_software.zip). Then, a hidden Markov model (HMM) was used to predict final DI-TADs based on the DI as implemented in DomainCaller. To obtain more comprehensive TAD information, we introduced another common TAD-calling algorithm (i.e., the IS algorithm). The IS reflects the aggregate of interactions passing across each bin, and IS boundaries were called using the public code (matrix2insulation.pl, https://github.com/dekkerlab/cworld-dekker) with parameters (−v -is 260,000 -ids 200,000 -im mean -nt 0.1 -bmoe 0) to divide the large DI-TADs. If an IS boundary was located within a DI-TAD and the insulation score of the IS boundary was lower than the average insulation score of the DI-TAD boundaries, the DI-TAD was divided into two smaller TADs. To compare the TAD structure between wild boar and Bama pig in each AT depot, we merged the raw Hi-C reads of each pair of replicates and thus determined common TADs for each AT depot. TAD boundaries were defined as breed-specific boundaries, as previously described [5], when boundary regions were called in only one breed and their DIs lacked significant correlation between wild boar and Bama pig compared to a random distribution of Spearman correlations. Specifically, for each boundary, we calculated Spearman’s correlation for the DI around the center of the boundary (+/− 10 bins) between breeds. Similarly, random Spearman’s correlation of DI was calculated by randomly selecting 20 bins from each breed, and the process was repeated 10,000 times to achieve a random distribution. The random correlation coefficients were translated into standardized z scores using the ‘scale’ function in R (version 3.6.1), and we assigned a *P* value to each z score using the ‘pnorm’ function in R (version 3.6.1). We selected the random correlation coefficient whose *P* value was 0.05 as the cutoff. If a boundary existed in only one breed and its Spearman correlation between the two breeds was lower than this cutoff, the boundary was defined as a breed-specific boundary.

### Identification of promoter-enhancer interactions (PEIs)

To identify promoter-enhancer interactions (PEIs), we pooled Hi-C reads from each pair of replicates for each AT depot and used the combined data in the analysis. First, we defined the regions from 2200 bp upstream to 500 bp downstream of the TSS as promoter regions. Overrepresented promoter-centered interactions were then called by the published PSYCHIC algorithm (https://github.com/dhkron/PSYCHIC) with default parameters as previously described [25] based on KR and quantile normalized intra-chromosomal observed contact matrices at 10-kb resolution. Briefly, topological domains were identified throughout the genome as implemented in the PSYCHIC algorithm, and the hierarchical domains were generated by merging similar neighboring domains. A domain-specific background model was then built for each domain or hierarchical domain according to the PSYCHIC algorithm. For promoter-centered interactions (±10 Mb), we compared the observed contact frequency (contact frequency in KR and quantile normalized contact matrices) with its expected contact frequency (defined as the observed contact frequency normalized to the domain-specific background model, output by PSYCHIC) to identify overrepresented interactions, and the statistical significance score (*P* values and FDR values) was output by PSYCHIC. To obtain high-confidence putative PEIs, we filtered the interactions between promoters (i.e., whether or not the two anchors of the interaction both contained promoters) and applied a hard cutoff with FDR value ≤ 0.001 and interaction length ≥ 50 kb.

### Calculation of regulatory potential score (RPS)

To assess the regulatory influence of enhancers on genes and thus accurately elucidate the dynamic rewiring of PEIs in wild boar and Bama pigs for each AT depot, we calculated the regulatory potential score (RPS) that was introduced in our previous study [12]. According to a biochemical assumption that enhancers’ contribution to the expression level of a specific gene is additive, the RPS is calculated as ∑n (log_10_ *I*_n_), in which *I*_n_ is the normalized interaction intensity (i.e., the observed contact frequency minus the expected contact frequency). The observed contact frequency of PEI was obtained from KR and quantile normalized contact matrices, and the expected contact frequency of PEI was obtained from expected contact matrices that were calculated using the domain-specific background model described above (output by PSYCHIC). If a gene does not form a PEI, the RPS = 0. To robustly identify genes associated with meaningful differences in RPS between Bama pigs and wild boar, we employ thresholds for both the minimum absolute fold change (|log_2_ fold change| > 1) and the minimum absolute difference (|delta| > 4); under this criterion in our study, the gene has lost or gained at least 3 PEIs (data not shown). This can exclude genes that show large fold changes but very small absolute differences in RPS.

### RNA-seq and expression analysis

To explore the relationship between gene expression and 3D genome conformation, we constructed four RNA-seq libraries for ATs in wild boars (two libraries for ULB and GOM, separately) and three libraries for ATs in Bama pigs (one library for ULB and two libraries for GOM). One corresponding library for ULB in Bama pigs was collected from our previous study [17]. Total RNA was extracted from each sample using an RNeasy Mini Kit (Qiagen, Valencia, CA, USA). Purified RNA was quantified by Nanodrop, and integrity was validated using an Agilent 2000. Strand-specific RNA-seq libraries were then generated using a rRNA depletion method (Globin-Zero Gold rRNA Removal Kit, Illumina, San Diego, CA, USA) coupled with a NEBNext® Ultra™ Directional RNA Library Prep Kit for Illumina® (NEB, Ipswich, MA, USA). All libraries were then sequenced on a HiSeq X Ten platform (Illumina) with a paired-end sequencing length of 150 bp. Kallisto (version 0.44.0) [26] was applied to align high-quality reads to the pig reference genome (*Sscrofa* 11.1) with default parameters, and the expression level of protein-coding genes was quantified as transcripts per million (TPM). The gene annotation file was downloaded from Ensembl Sscrofa 11.1 (Release 102). We next analyzed the differentially expressed genes (DEGs). For each adipose depot comparison, genes expressed in at least one breed (TPM > 0.1 for both replicates) are used for subsequent analysis and read counts of each gene obtained from Kallisto (version 0.44.0) served as input values. The R package “DESeq2” (version 1.28.1) [27] was applied using the default parameters to identify genes that are differentially expressed in wild boars and Bama pigs. Significant DEGs were defined using the criteria FDR < 0.05 and |log_2_ fold change| > 1 (Additional file 8: Table S7).

For RNA-seq data visualization, bam files were generated using STAR software [28] (version 2.6.0c) with default parameters to map read pairs to pig reference genome, and then bam files were converted to big wigs for visualization using bamCoverage software [29] (version 3.5.0) with parameters (−-normalizeUsing RPKM --binSize 10 -p 4).

### Functional enrichment analysis

Functional enrichment analysis of specific gene sets was performed using Metascape (http://metascape.org) [30] with default parameters. Specifically, pig genes were converted to their human orthologous, and humans (*Homo sapiens*) were selected as the target analysis species. In the compartment comparison, all genes in the genome served as background. In the PEI comparison, we selected genes that showed consistent changes in expression and RPS to perform functional enrichment analysis. Thus, a subset of expressed genes (at least one breed in the comparison has TPM > 0.1) was used as background in the functional enrichment analysis. Gene Ontology (GO)-biological processes (GO-BP) and Kyoto Encyclopedia of Genes and Genomes (KEGG) pathways were used as ontology sources. The most statistically significant terms in each cluster are shown.

### Statistical analyses

All statistical analyses were performed using the ‘wilcox.test’ (Wilcoxon rank-sum test) or ‘prop.test’ function (two proportions test) in R (version 3.6.1).

## Results

### Hi-C data description

**Fig. 1:**
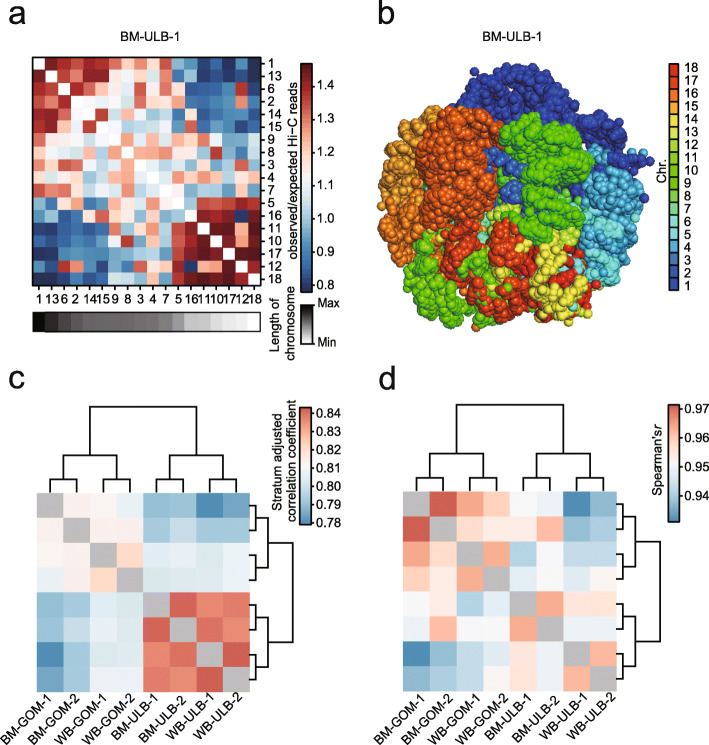
Overview of 3D genome organization in wild boar and Bama pig adipose tissues. (**a**) Example of observed/expected contact matrices between 18 autosomes in Bama pig (BM) ULB-1 at 500-kb resolution. Chromosomes with similar lengths are spatially close. (**b**) Simulated structure of 3D genome organization in BM-ULB-1. (**c**) Estimated interrelationship between intra-chromosomal Hi-C maps of wild boar (WB) and BM adipose tissues (ATs) at 100-kb resolution. (**d**) Spearman’s correlation coefficient heatmap of gene expression comparisons between pairwise samples

To comprehensively explore the reorganization of the chromatin architecture of ATs between wild boars and Bama pigs, we analyzed an in situ high-throughput chromatin conformation capture (Hi-C) map of representative subcutaneous adipose depots (i.e., in the upper layer of backfat, ULB) and visceral adipose depots (i.e., in the greater omentum, GOM) in two adult female wild boars and Bama pigs (Additional file 2: Table S2). A total of ~ 2.5 billion valid contacts were obtained from eight libraries with a depth of ~ 319 million (M) contacts per library and a maximum resolution of ~ 7.1 kb (Additional file 3: Fig. S1a; Additional file 2: Table S2). The contact quality of the Hi-C data was validated by the *cis*/*trans* interaction ratio (~ 68.07% *cis* contacts), the interaction distance (~ 64.63% *cis* contacts over 20 kb), and the distance-dependent decay of *cis* contact frequencies (Additional file 3: Fig. S1b-d; Additional file 2: Table S2). Based on these contact data, chromatin conformation was plotted as the chromatin interaction frequency. The plots revealed the presence of territorial architectures in the pig genome (Fig. 1a). Additionally, 3D modeling of the pig genome clearly showed the spatial relationships between genomic regions, further supporting the chromosome territories observed in the pig genome (Fig. 1b). To assess the similarity of chromatin architecture in different samples, we generated normalized intra-chromosomal contact maps using the method of KR [2] and the quantile algorithm [20] at 100-kb resolution (~ 99.83% of bins in 100 kb having at least 1000 intra-chromosomal contacts) (Additional file 3: Fig. S1a). The global contact maps obtained for replicates were highly reproducible as determined by HiCRep [21] (median stratum-adjusted correlation coefficient [SCC] = 0.83), and the contact maps for the same adipose depots were also highly similar between wild boars and Bama pigs (median SCC = 0.82) (Fig. 1c). Hierarchical clustering of the RNA-seq data recapitulated these findings (Fig. 1d). These results suggest that there are limited changes in global chromatin organization and in the transcriptome between wild boar and Bama pig ATs. Given that the 3D genome is organized into hierarchical layers and that previous literature have reported increasing numbers of changes as one descends the genome architectural hierarchy [4, 31], we next performed comparative Hi-C analyses of ATs in wild boars and Bama pigs at various chromatin scales.

### Dynamic chromatin compartmentalization in wild boar and Bama pig ATs

We first identified large compartment intervals at 500 kb resolution, which represents subchromosomal organization and is closely linked to euchromatin and heterochromatin [3, 32]. For each sample, we identified ~ 327 compartment A intervals (the number of such regions ranged from 310 to 340, with a median size between 2 and 2.5 Mb) and ~ 332 compartment B intervals (the number of such regions ranged from 320 to 342, with a median size between 2 and 2.5 Mb) (Additional file 4: Table S3). Given that a previous study showed that precise A/B compartments (also termed as compartmental domains) can be observed at high resolution (e.g., 10-kb) [23]. To make a more precise comparison between two pig breeds, we further segregated the genome into small compartment A (an average of 1.12 Gb or 49.67% of the genome) and compartment B (an average of 1.14 Gb or 50.33% of the genome) regions at 20-kb resolution (~ 99.00% of bins in 20-kb having at least 1000 intra-chromosomal contacts) (Additional file 3: Fig. S2a, Additional file 4: Table S3). As expected, the compartment A regions were enriched in GC content and protein-coding genes and showed higher levels of gene expression than the compartment B regions (*P* value < 10^−16^ for each comparison, Wilcoxon rank-sum test) (Additional file 3: Fig. S2b). To ensure reliability, we also identified compartments at 100-kb resolution, a level of resolution that is commonly used in compartment calling. Overall, a good correspondence was observed when we performed comparisons of compartment calls made at 20, 100 and 500 kb resolution (~ 87.68% of A/B compartments at 20 kb were assigned the same compartment type at 100 and 500 kb in each sample) (Additional file 3: Fig. S2c). These observations suggesting reliability of the A/B compartment assignments at 20 kb resolution.

**Fig. 2:**
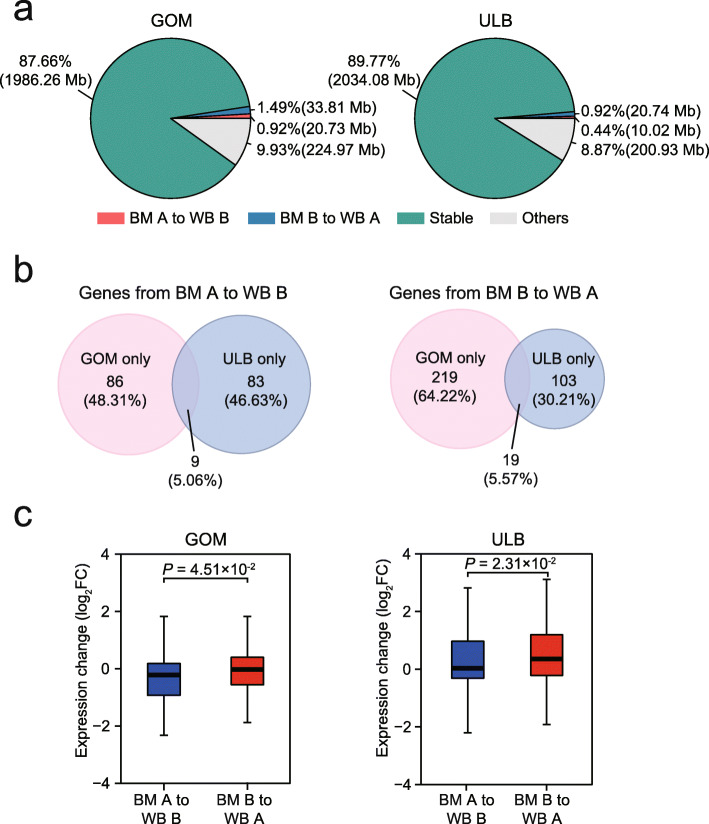
Changes in compartment A/B across wild boar and Bama pig ATs. (**a**) Percentage of stable or switched compartments between WB and BM ATs in the genome. Genomic regions where compartment changes are inconsistent in all pairs of replicates are defined as others. (**b**) Overlap of compartment-switched genes between the ULB (from BM to WB, 92 genes from A to B, 122 genes from B to A) and the GOM (95 genes from A to B, 238 genes from B to A). (**c**) Changes in the expression of genes whose compartment status was switched between WB and BM ATs. *P* values were calculated using the Wilcoxon rank-sum test

Comparing the compartment states in wild boar with those in Bama pigs for each AT revealed that ~ 3.66% of the genome (82.90 Mb) underwent compartment switching in at least one AT type (Additional file 4: Table S3). Notably, we observed more A/B compartment switching in the GOM (54.54 Mb, 2.41% of the genome) than in the ULB (30.76 Mb, 1.36% of the genome) (*P* value = 0.013, two proportions test) (Fig. 2a), reflecting that the GOM underwent more such alterations among wild boars and Bama pigs. Moreover, the genes in specific compartment switching regions in the ULB (from Bama pig to wild boar, 92 genes changed from A to B, 122 genes from B to A) differed drastically from those in the GOM (95 genes from A to B, 238 genes from B to A) (genes from A to B and from B to A have 5.06% and 5.57% overlaps between ULB and GOM, respectively), implying that the AB switching that occurs in the ULB and that that occurs in the GOM are associated with different biological functions (Fig. 2b).

**Fig. 3:**
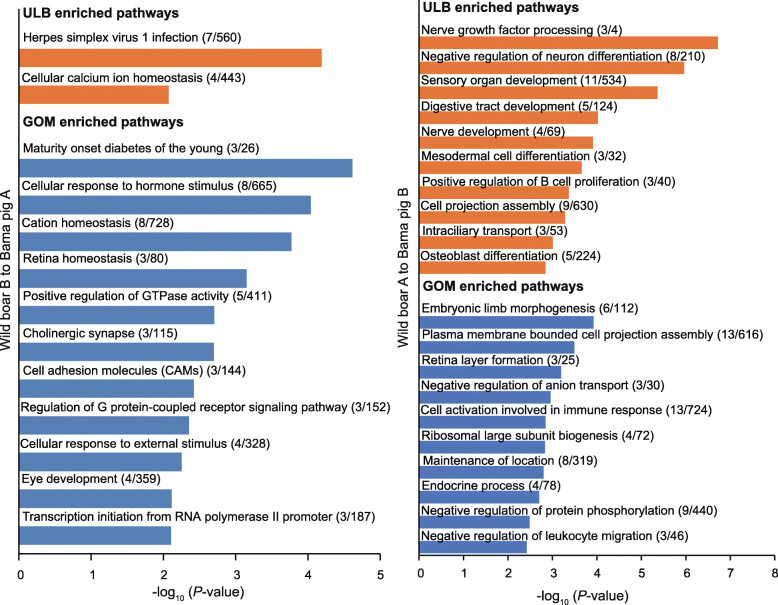
Functional enrichment analysis of the genes displaying compartment switching between WB and BM in each AT

**Fig. 4:**
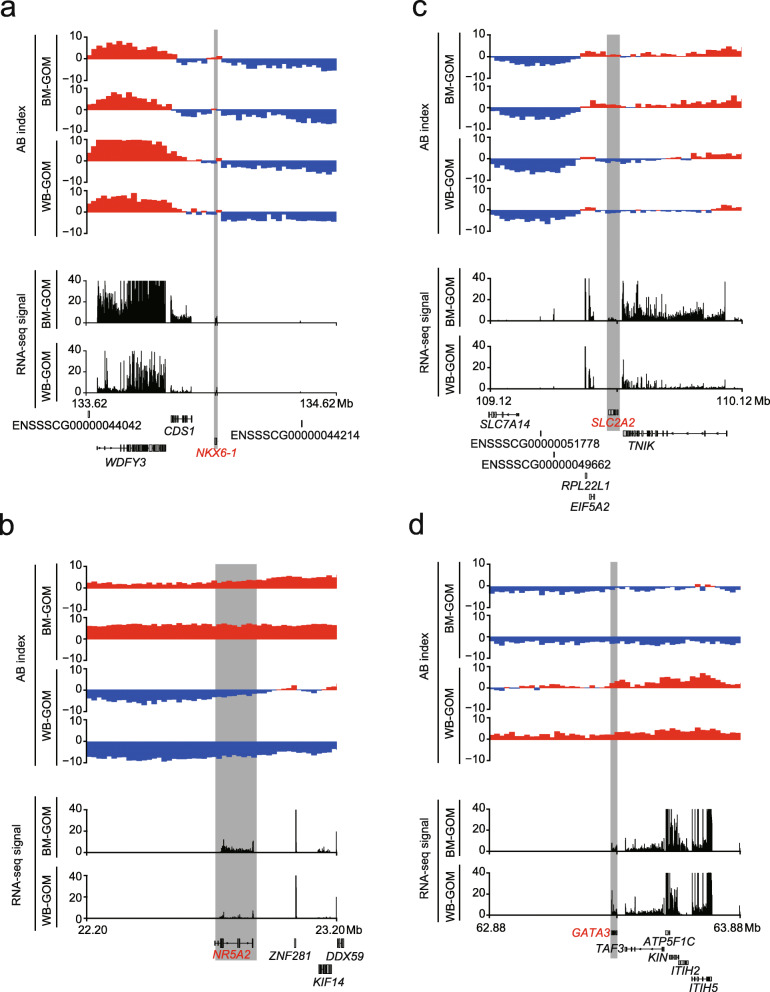
Example of genes associated with compartment switching. (**a-d**) Genome browser view of the compartmentalization pattern and expression level of *NKX6–1* (**a**)*, NR5A2* (**b**), *SLC2A2* (**c**) and *GATA3* (**d**) between WB and BM GOM. Top panel: AB index pattern in each Hi-C library. Bottom panel: RNA-seq profiles, the RNA-seq signal represents the number of reads mapped to each genomic region. The gray area represents the location of the target gene

We then examined the genes within these compartment switching regions for each AT depot. As expected, genes that switched from B to A from Bama pig to wild boar (*n* = 122 in the ULB; *n* = 238 in the GOM) tended to show increased expression compared with genes that switched from A to B (*n* = 92 in the ULB, *n* = 95 in the GOM). (Fig. 2c). Functional enrichment showed that genes associated with the Bama pig-restricted compartment A in the ULB were significantly enriched in only two pathways, ‘herpes simplex virus 1 infection’ and ‘cellular calcium ion homeostasis’ (Fig. 3). Genes changed in the GOM are associated with a broad spectrum of biological functions, including metabolism, homeostasis, cellular response to stimulus, G protein-coupled receptor signaling pathway, development and regulation of gene transcription (Fig. 3). Of these, the metabolism-related pathway ‘maturity-onset diabetes of the young’ was the top enriched term. Three genes associated with this pathway, *NKX6–1*, *NR5A2* and *SLC2A2*, showed increased expression in Bama pig GOM compared with wild boar (Fig. 4a-c), highlighting their potential as novel interesting candidates for further functional characterization.

Next, we analyzed the function of genes associated with wild boar-restricted compartment A. We found that genes changed in the ULB were enriched in pathways related to development (e.g., ‘sensory organ development’, ‘digestive tract development’), regulation of neural growth and differentiation (e.g., ‘nerve growth factor processing’ and ‘negative regulation of neuron differentiation’) and the immune response (e.g., ‘positive regulation of B cell proliferation’) (Fig. 3). In the GOM, we observed that wild boar-restricted compartment A regions were also enriched in genes related to the immune response, including ‘cell activation involved in immune response’ and ‘negative regulation of leukocyte migration’ (Fig. 3). Typically, GATA binding protein 3 (GATA3) (Fig. 4d) acts as a master of many trades in immune regulation [33]. In addition, genes associated with basic cellular functions (e.g., negative regulation of anion transport, ribosomal large subunit biogenesis and negative regulation of protein phosphorylation), morphogenesis and endocrine processes were also enriched in wild boar-restricted compartment A regions in the GOM.

### Comparison of TAD structure in ATs in wild boars and Bama pigs

**Fig. 5:**
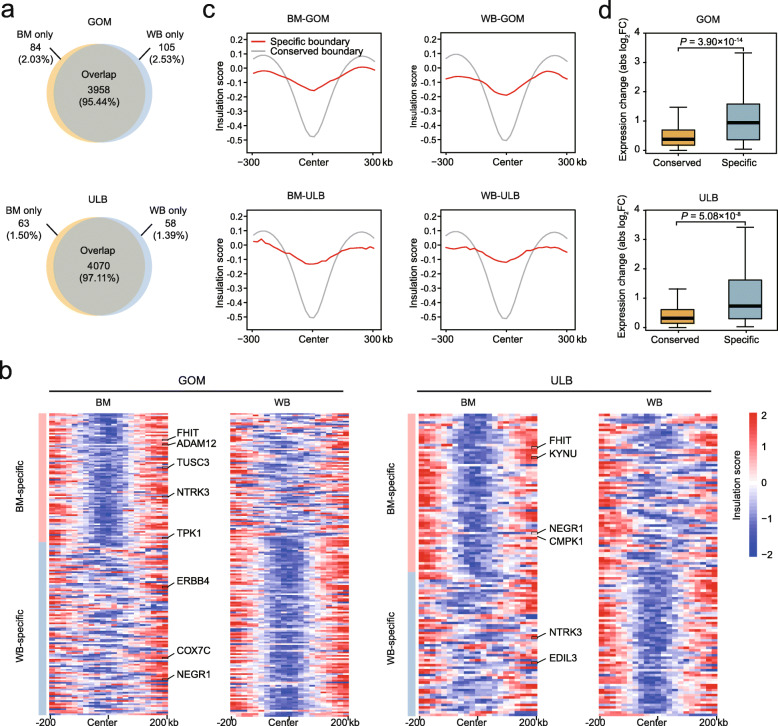
TAD boundaries are largely stable in wild boar and Bama pig ATs. (**a**) Overlap of TAD boundaries in WB and BM ATs. (**b**) Average insulation scores in a 400-kb region centered on breed-specific boundaries of WB and BM ATs. (**c**) Average insulation score between breed-conserved and breed-specific boundaries in each AT. (**d**) Absolute value of expression change for genes localized in breed-conserved (*n* = 11,095 in GOM, *n* = 10,841 in ULB) or breed-specific TADs (*n* = 109 in GOM, *n* = 73 in ULB). *P* values were calculated using the Wilcoxon rank-sum test

At a finer scale, the mammalian genome is partitioned into TADs that serve as fundamental regulatory chromatin structures and are largely conserved across tissues and species [5]. To understand how TADs in adipose depots differ in the two pig breeds, we defined TADs using a DI [5] and IS [24] algorithms at 20-kb resolution. These values were highly reproducible in the biological replicates (Additional file 3: Fig. S3a) (Spearman’s *r* (DI) > 0.93; Spearman’s *r* (IS) > 0.97); therefore, we merged the replicates and defined an average of 3845 (3682 to 3942) TADs in each AT. The numbers and sizes of TADs in both the ULB and the GOM were similar in wild boars and Bama pigs (Additional file 3: Fig. S3b, c). We found that the TAD boundaries enriched genes compared to the flanks (Additional file 3: Fig. S3d) and the contact frequency within the TAD was higher than that outside (Additional file 3: Fig. S3e). These findings are in line with the general characteristics of TADs [5] and indicate the reliability of our TADs. We then performed cross-breed comparisons and found that the majority of TAD boundaries (~ 97.11% in the ULB, ~ 97.03% in the GOM) were conserved and that only a small portion of boundaries were gained or lost as wild boar- or Bama pig-specific boundaries (121 [2.89%] in the ULB and 189 [2.97%] in the GOM) (Fig. 5a, b; Additional file 5: Table S4). Comparison of the features of conserved and breed-specific TAD boundaries showed that conserved boundaries have stronger insulation in all adipose depots (Fig. 5c).

Finally, we examined the genes in breed-specific TADs and conserved TADs. We found that genes within breed-specific TADs showed significant differences in expression between wild boar and Bama pigs compared with genes in conserved TADs (Fig. 5d), suggesting that reconstruction of TADs between pig breeds affects gene expression. In the comparison between pig breeds, we identified 33 and 43 expressed genes (gene expressed in at least one breed [TPM > 0.1 for both replicates]) within breed-specific TADs with clear gene symbol in the Ensembl database (Release 102) in the ULB and the GOM, respectively. Some of these genes, such as *NEGR1*, *EDIL3*, *CMPK1*, *COX7C*, *FHIT*, *TPK1*, *ADAM12* and *ERBB4*, are related to metabolic pathways (Fig. 5b).

### Rewiring of promoter-enhancer interactions (PEIs) in ATs in wild boars compared to Bama pigs

**Fig. 6:**
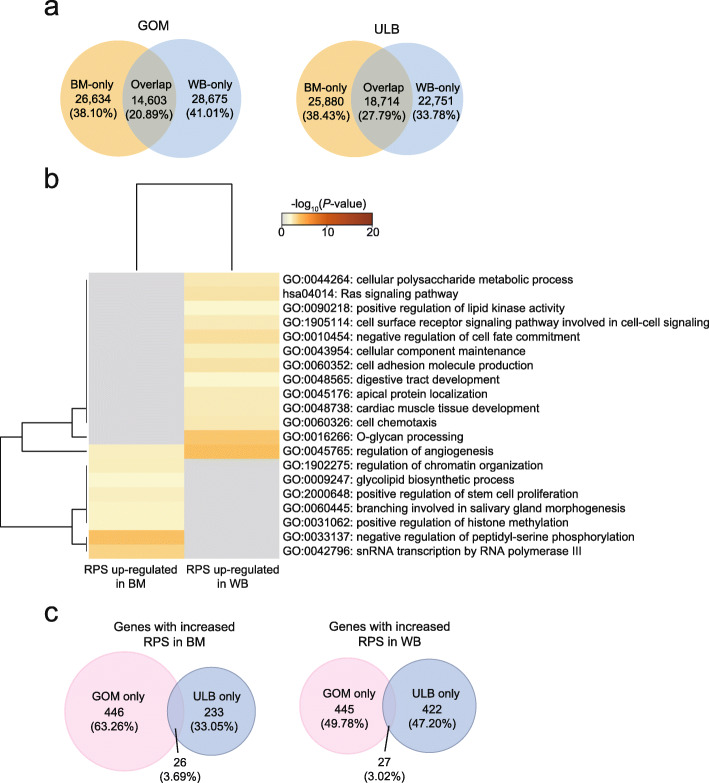
PEI rewiring in wild boar and Bama pig ATs. (**a**) Overlap of PEIs between WB and BM ATs. (**b**) Functional enrichment of genes that associated with meaningful differences in RPS between Bama pigs and wild boar. (**c**) Overlap between the ULB and the GOM in genes with meaningful changes in RPS

Compared to TADs, PEIs are more dynamic across tissues and developmental stages and are thought to be closely associated with the regulation of gene expression [34, 35]. We identified ~ 42,644 PEIs in each AT at 10-kb resolution using PSYCHIC (replicates were merged and reached ~ 98.74% of the 10-kb bins that had at least 1000 reads), overlapping ~ 14,733 genes (Additional file 6: Table S5). More than 70% of PEIs were restricted to TADs, and more than 60% of PEIs bypassed the closest putative enhancer to interact with more distal *cis*-regulatory elements (Additional file 3: Fig. S4a, b). Gene expression was positively correlated with the number of PEIs (Additional file 3: Fig. S4c). We also used our previously introduced regulatory potential score (RPS) to explore the regulatory effects of PEIs on individual genes [12] and found that increased gene expression was associated with enhanced RPS (Additional file 3: Fig. S4d). These findings demonstrate the accuracy of PEI calling in our study. In contrast to the largely conserved A/B compartments and TAD structures, most PEIs were extensively rewired between wild boar and Bama pig ATs (Fig. 6a).

To understand how the extensive rewiring of PEIs may contribute to changes in the transcriptome, we compared the RPS for each gene and identified genes associated with meaningful differences in RPS between wild boar and Bama pig. After combining PEI rewiring events, a set of 787 genes (368 in the ULB, 419 in the GOM) whose changes in expression were consistent with the observed RPS changes were identified (Additional file 7: Table S6). Of these, 371 genes engaged in more interactions in wild boars than in Bama pigs, and this was followed by increased gene expression, while 416 genes exhibited fewer interactions in wild boars than in Bama pigs, and these genes were expressed at lower levels in wild boars than in Bama pigs. Functional enrichment analysis showed that genes with increased RPS and expression level in Bama pigs were enriched in categories related to basic cellular functions, (e.g., ‘regulation of chromatin organization’, ‘positive regulation of histone methylation’, ‘snRNA transcription by RNA polymerase III’,' negative regulation of peptidyl-serine phosphorylation’), glycolipid biosynthesis, angiogenesis and stem cell proliferation (Fig. 6b). Genes with increased RPS and expression level in wild boars were associated with energy metabolism (e.g., ‘cellular polysaccharide metabolic process’, ‘Ras signaling pathway’, ‘positive regulation of lipid kinase activity’), angiogenesis, cell-cell signaling, development and immune responses (e.g., ‘cell adhesion molecule production’ and ‘cell chemotaxis’) (Fig. 6b).

**Fig. 7:**
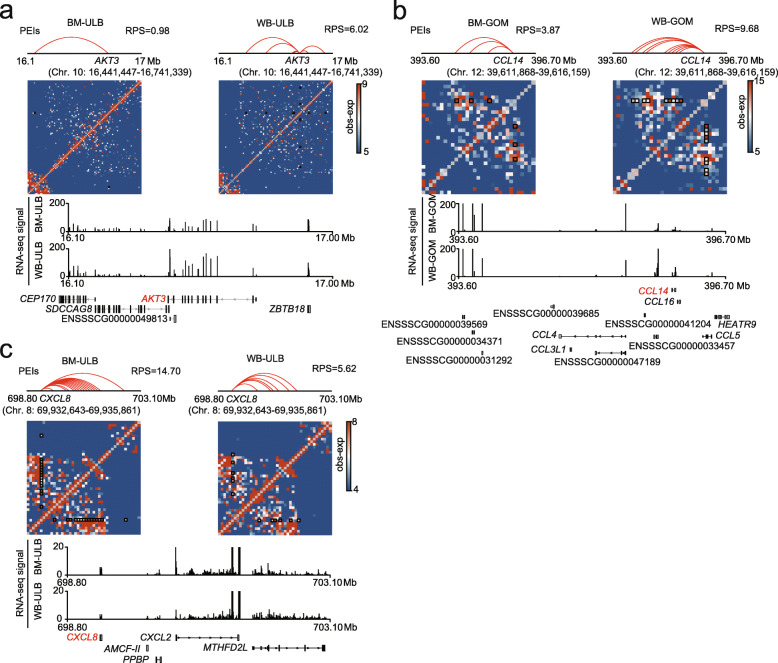
Example of genes associated with meaningful differences in RPS between wild boar and Bama pig ATs. (**a-c**) Schematic representation of the PEIs of *AKT3* (**a**), *CCL14* (**b**) and *CXCL8* (**c**). Top panel: Schematic diagram of the PEIs for target gene. Genomic regions under the gene name represents the precise gene position. Middle panel: Hi-C contact map around the target gene. The pixel enclosed by the black square is PEI. Bottom panel: RNA-seq profiles, which represents the reads signal obtained by RNA sequencing

Notably, similar to A/B compartment switching, most of these concordant genes differed in an AT-type-specific manner (Fig. 6c). Therefore, we further compared the functions of these AT-specific altered genes. For the genes with high RPS in wild boars, we observed functional heterogeneity between ULB-specific and GOM-specific changed genes. We found that the genes with a specific high RPS in the ULB were enriched in a few categories, including ‘regulation of epithelial cell proliferation’ and ‘p53 signaling pathway’ (Additional file 3: Fig. S5a). Previous studies have shown that p53 signaling is involved in the negative regulation of adipogenesis. For example, *AKT3*, which can inhibit adipogenesis [36], specifically exhibited higher RPS and higher expression levels in wild boar ULB than in Bama pigs (Fig. 7a). Genes expressed in the GOM with a specific high RPS were related to angiogenesis, cell differentiation (e.g., ‘negative regulation of cell fate commitment’ and ‘glial cell differentiation’), energy metabolism (e.g., ‘regulation of kinase activity’ and ‘positive regulation of lipid kinase activity’), and morphogenesis (e.g., ‘tissue morphogenesis’) (Additional file 3: Fig. S5a). In addition, we observed that GOM-specific changed genes were also overrepresented in categories associated with the immune system (e.g., ‘response to wounding’, ‘positive regulation of apoptotic process’, ‘endocytosis’ and ‘cell chemotaxis’). Typically, expression of *CCL14,* a gene that encodes a chemokine, can promote the activation of immune cells [37, 38] (Fig. 7b).

For the genes with high RPS in Bama pigs, we found that those in the GOM with a specific high RPS were only enriched in basic cellular functions (‘negative regulation of histone modification’) (Additional file 3: Fig. S5b). The genes in the ULB with specific high RPS were related not only to basic cellular functions (e.g., ‘negative regulation of peptidyl-serine phosphorylation’ and ‘regulation of chromatin organization’) but also to disease and immune responses (e.g., ‘regulation of necrotic cell death’, ‘pathogenic *Escherichia coli* infection’, ‘regulation of viral process’) (Additional file 3: Fig. S5b); one example of such a gene is *CXCL8* (also known as *IL-8*) [39] (Fig. 7c).

## Discussion

### A resource for studying the chromatin organizations of multiple porcine adipose depots in different pig breeds

Previous studies have shown that ATs can be anatomically categorized into subcutaneous adipose tissue (SAT) and visceral adipose tissue (VAT); these ATs differ in structural organization, cellular size, and biological function [40, 41]. To provide comprehensive insight into the organization of chromatin in porcine adipose tissue, we sampled one representative subcutaneous adipose depot (ULB) and one visceral adipose depot (GOM) in each of two pig breeds (i.e., Bama pig and wild boar) and performed a Hi-C study of the sampled material.

Several previous studies [10–13, 17] have characterized the architecture of chromatin in porcine tissues and cell types. These studies mainly compared chromatin organization between species or during early development, and limited data on the differences in chromatin organization between pig breeds were presented. Thus, our study provides a resource to fill this gap to a certain extent. Additionally, our Hi-C data also allowed the investigation about the specific regulation of the different adipose depots.

### Analysis of compartments across pig breeds

In this study, we identified compartment intervals at 500-kb resolution. The number and length of these intervals are similar to those in muscles of pig fetuses [13] or in pig liver cells at 500-kb resolution [10]. Additionally, we also identified small A/B compartments at 20-kb resolution, which termed as compartmental domains in previous study [23]. These small compartments provide a more precise annotation of A/B status in the pig genome, and can be used as a resource for studying the fine regulation effect of A/B compartment on genes in pigs.

We observed that 1.36% (ULB) to 2.41% (GOM) of the genomic regions represented compartment states that were switched between the two pig breeds. These percentages are lower than the percentage previously reported for compartment switching during the development of porcine muscle from 35 to 80 days of gestation (~ 11.43%) [12] and slightly lower than the percentage reported for porcine muscle from 90 to 110 days of gestation (~ 3%) [13]. Our findings suggest that A/B compartments are mostly conserved between adult Bama pigs and wild boars. Notably, the genes within compartment-switched regions between Bama pigs and wild boars were more likely to be depot-specific, reflecting the complexities of ATs from the perspective of 3D genome architecture.

### TADs are stable between Bama pigs and wild boars

In addition to A/B compartments, TAD boundaries in different adipose depot types are largely conserved between the two pig breeds, consistent with the previously reported stability of TADs across different cell types and species [5]. Previous studies have shown that alterations in TAD structure can lead to the acquisition or loss of precise architecture (e.g., PEI) related to gene regulation [42]. We found that the expression of genes within breed-specific TADs is more dynamic than that of genes within conserved TADs, supporting the idea that TADs have a regulatory role. Additionally, we found that conserved TAD boundaries have higher insulation strengths than breed-specific boundaries in all adipose depots, implying an association between boundary stability and insulation strength. Similar results were obtained in a previous cross-species comparative study [10]. This finding suggests that insulation strength has the potential to predict the stability of TAD boundaries.

### Extensive reorganization of PEIs between Bama pigs and wild boars

Hi-C requires deep sequencing to characterize PEIs. To increase the detection power of the method, we merged replicates and used the combined data to identify PEIs at 10-kb resolution. Using a series of strict criteria in the PSYCHIC algorithm, we identified ~ 42,644 PEIs for each adipose depot, indicating that enhancers are universal regulatory elements in the pig genome. The expression levels of genes were positively correlated with the number of enhancers, in agreement with the finding that enhancer effects on target gene transcription are additive [43, 44]. Many enhancers often ‘skip’ nearby genes in favor of interactions with more distally located genes [45–47]. Our constructed PEI profiles that assign putative enhancers to gene promoters may provide a resource for studying long-range gene regulation of pig ATs.

Compared with TADs and compartments, the promoter–enhancer interactome is more dynamic between the two studied pig breeds, consistent with the idea that chromatin structure may perhaps be more extensively reorganized locally [48]. Furthermore, the PEIs that are rewired between wild boar and Bama pigs may also provide a candidate set of *cis*-regulatory elements that may contribute to phenotypic differences between the breeds.

Dynamic PEIs are widely involved in biological functions. In addition to terms related to adipose-specific functions (e.g., energy metabolism), some pathways related to basic cell functions also showed significant enrichment. This highlights the complexity of function of the adipose depots of Bama pigs and wild boars. Moreover, the genes associated with PEIs that were differently organized in the two pig breeds differed between the GOM and the ULB. For example, genes related to the p53 signaling pathway were associated with specifically remodeled PEI structures in the ULB in the two pig breeds. Given that p53 signaling can negatively regulate adipogenesis [49, 50], this finding implies that these changes may be related to the difference in backfat phenotype between Bama pigs and wild boars. Hence, our results also provide information that increases the current understanding of the roles of different adipose depots in various pig breeds.

## Conclusions

We report here a comparative analysis of three-dimensional genome organization in the fat depots of Bama pigs and wild boars. In a hierarchical model of chromatin organization, large chromatin structures, including A/B compartments and TADs, are mostly conserved between Bama pigs and wild boars. Conversely, PEIs have been widely rewired, indicating that PEIs have greater potential to regulate the phenotypic differences between pig breeds. Differences in chromatin organization among pig breeds are associated with fine-tuning of gene expression levels and involve genes with a broad spectrum of biological functions. Additionally, these changes in chromatin structure among breeds are heterogeneous between the GOM and the ULB. These findings provide an understanding of how the chromatin architecture in adipose depots differs among pig breeds. Our study can also be used as a resource to decipher the regulatory mechanisms that result in phenotypic differences among pig breeds.

## Supplementary Information

**Additional file 1: Table S1.** Nutrient levels in the experimental diets.

**Additional file 2: Table S2.** Sample and data information for Hi-C.

**Additional file 3: Fig. S1.** Evaluation of Hi-C data in wild boar and Bama pig adipose tissues. (a) Hi-C map resolutions at various bin sizes. The maximum resolution was defined as the smallest bin size at which 80% of loci had at least 1000 intra-chromosomal contacts. (b) Ratio of *cis* to *trans* interactions in total valid contacts. (c) Proportions of long-distance (> 20 kb) components in *cis* interactions. (d) Log-log contact frequency as a function of genomic distance. **Fig. S2.** Characteristics of compartments A/B. (a) Proportions of A and B compartments in each sample. (b) Genomic features (GC content, gene density and gene expression) in compartments A and B in each sample. *P* values were calculated by Wilcoxon rank-sum test. The number represents the population size (20 kb bins) in comparison. (c) The percentage of genomic regions shows stable or changed compartment states between different resolutions in each sample. **Fig. S3.** Characterization of TADs in wild boar and Bama pig ATs. (a) Spearman’s correlation heatmap of the directionality index (DI) and insulation score (IS) between WB and BM ATs. (b) Bar plot showing the TAD number in each AT. (c) Distribution of TAD size in each AT. The horizontal line represents median TAD size, boxes indicate the 25th and 75th percentiles, and whiskers correspond to the 1.5× interquartile range. The median TAD size in each AT is shown above the boxplot. (d) The number of PCGs surrounding boundary regions in each AT. (e) Log-log contact frequency as a function of the genomic distance (≤ 2 Mb) within TAD and out of TAD in each AT. **Fig. S4.** Characterization of PEIs. (a) Percentage of PEIs located within or across TADs. (b) Percentage of promoters that interact with the nearest putative enhancers (no-skipping) and the percentage of those that skip at least one enhancer (skipping). (c) Expression level of genes with different interacting enhancer numbers in each AT. *P* values were calculated using the Wilcoxon rank-sum test. (d) Expression of genes in different RPS categories in each AT. *P* values were calculated using the Wilcoxon rank-sum test. **Fig. S5.** Function of genes with ULB-specific or GOM-specific RPS changes cross wild boar and Bama pigs.

**Additional file 4: Table S3.** A/B compartments in each adipose depot.

**Additional file 5: Table S4.** Conserved and breeds-specific TAD boundary positions.

**Additional file 6: Table S5.** Identified PEIs in each adipose depot.

**Additional file 7: Table S6.** Genes with covariation between expression and RPS.

**Additional file 8: Table S7.** Differentially expressed genes between two pig breeds.

## Data Availability

The datasets generated or analysed during this study are included in this published article are available from the Gene Expression Omnibus with the accession number GSE183447, GSM4942849 and GSM4943208.

## Abbreviations

ATs: Adipose tissues
BM: Bama pig
DI: Directionality index
GO: Gene Ontology
GOM: Greater omentum
Hi-C: High-throughput chromatin conformation capture
IS: Insulation score
KEGG: Kyoto Encyclopedia of Genes and Genomes
PEIs: Promoter-enhancer interactions
RPS: Regulatory potential score
SCC: Stratum-adjusted correlation coefficient
TADs: Topologically associating domains
TPM: Transcripts per million
ULB: Upper layer of backfat
WB: Wild boar
