# Supplementary material for: Reorganization of 3D genome architecture across wild boar and Bama pig adipose tissues
Source: J Anim Sci Biotechnol. 2022 Mar 12;13:32. doi: 10.1186/s40104-022-00679-2 (PMC8917667; doi:10.1186/s40104-022-00679-2)
Supplement: Supplementary file 3 — Additional file 3: Fig. S1. Evaluation of Hi-C data in wild boar and Bama pig adipose tissues. (a) Hi-C map resolutions at various bin sizes. The maximum resolution was defined as the smallest bin size at which 80% of loci had at least 1000 intra-chromosomal contacts. (b) Ratio of cis to trans interactions in total valid contacts. (c) Proportions of long-distance (> 20 kb) components in cis interactions. (d) Log-log contact frequency as a function of genomic distance. Fig. S2. Characteristics of compartments A/B. (a) Proportions of A and B compartments in each sample. (b) Genomic features (GC content, gene density and gene expression) in compartments A and B in each sample. P values were calculated by Wilcoxon rank-sum test. The number represents the population size (20 kb bins) in comparison. (c) The percentage of genomic regions shows stable or changed compartment states between different resolutions in each sample. Fig. S3. Characterization of TADs in wild boar and Bama pig ATs. (a) Spearman’s correlation heatmap of the directionality index (DI) and insulation score (IS) between WB and BM ATs. (b) Bar plot showing the TAD number in each AT. (c) Distribution of TAD size in each AT. The horizontal line represents median TAD size, boxes indicate the 25th and 75th percentiles, and whiskers correspond to the 1.5× interquartile range. The median TAD size in each AT is shown above the boxplot. (d) The number of PCGs surrounding boundary regions in each AT. (e) Log-log contact frequency as a function of the genomic distance (≤ 2 Mb) within TAD and out of TAD in each AT. Fig. S4. Characterization of PEIs. (a) Percentage of PEIs located within or across TADs. (b) Percentage of promoters that interact with the nearest putative enhancers (no-skipping) and the percentage of those that skip at least one enhancer (skipping). (c) Expression level of genes with different interacting enhancer numbers in each AT. P values were calculated using the Wilcoxon rank-sum test. (d) Expres [file 40104_2022_679_MOESM3_ESM.docx]

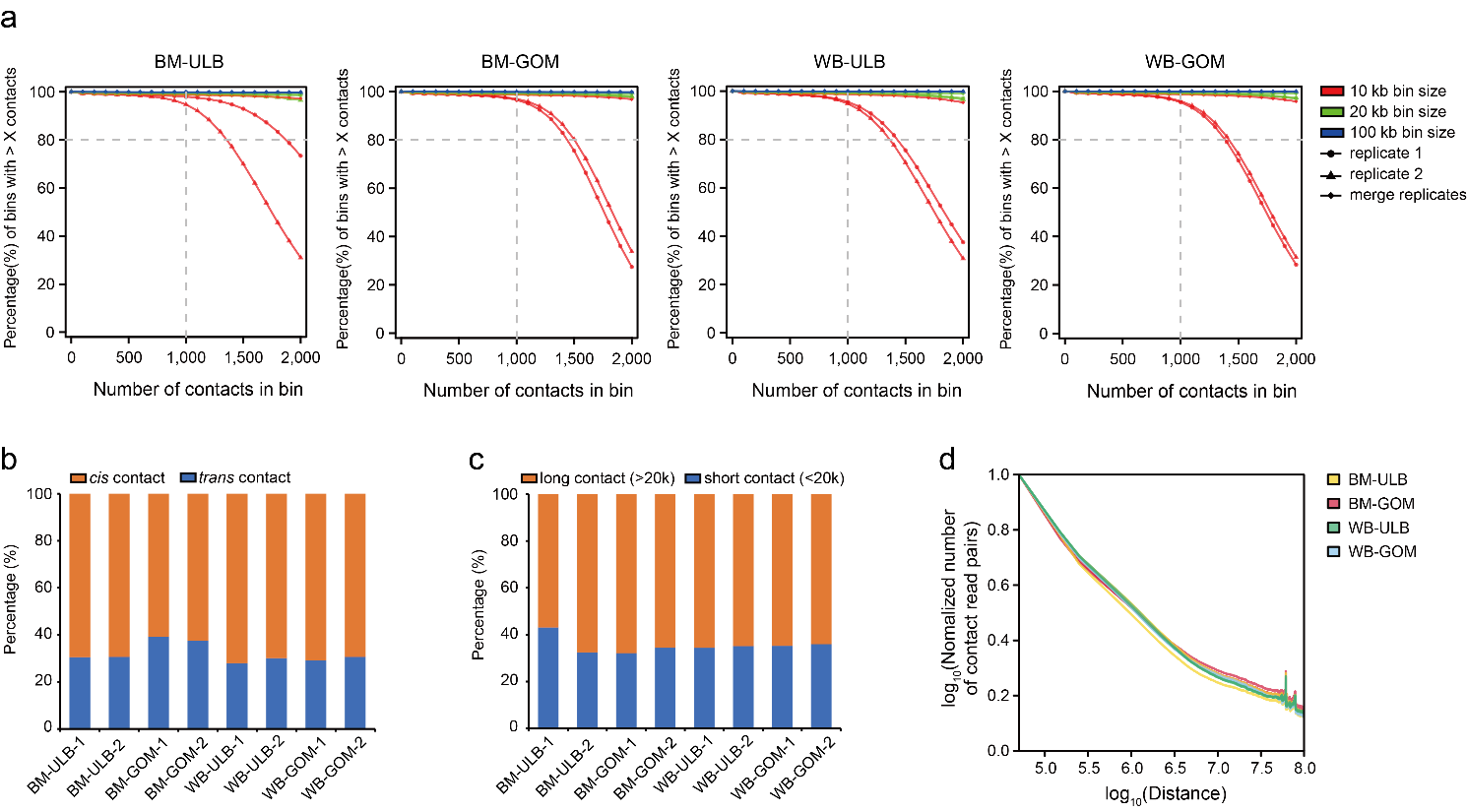


**Fig. S1 Evaluation of Hi-C data in wild boar and Bama pig adipose tissues.**

(a) Hi-C map resolutions at various bin sizes. The maximum resolution was defined as the smallest bin size at which 80% of loci had at least 1000 intra-chromosomal contacts. (b) Ratio of *cis* to *trans* interactions in total valid contacts. (c) The proportions of long-distance (> 20 kb) components in *cis* interactions. (d) Log-log contact frequency as a function of genomic distance.


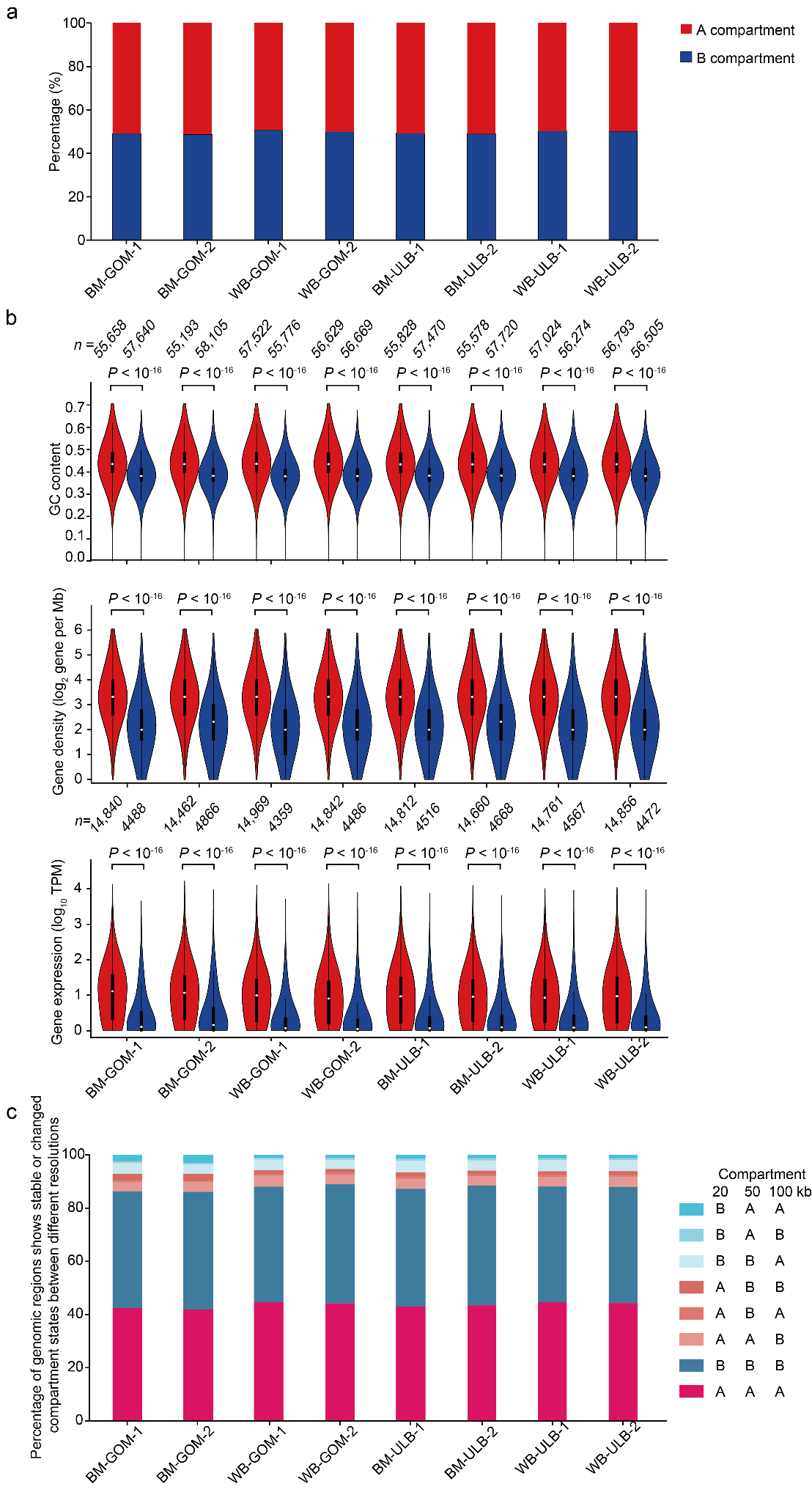


**Fig. S2 The characteristics of compartments A/B**

(a) The proportions of the A and B compartments in each sample. (b) Genomic features (GC content, gene density and gene expression) between compartments A and B in each sample. *P* values were calculated by Wilcoxon rank-sum test. The number represents the population size (20 kb bins) in comparison. (c) The percentage of genomic regions shows stable or changed compartment states between different resolutions in each sample.


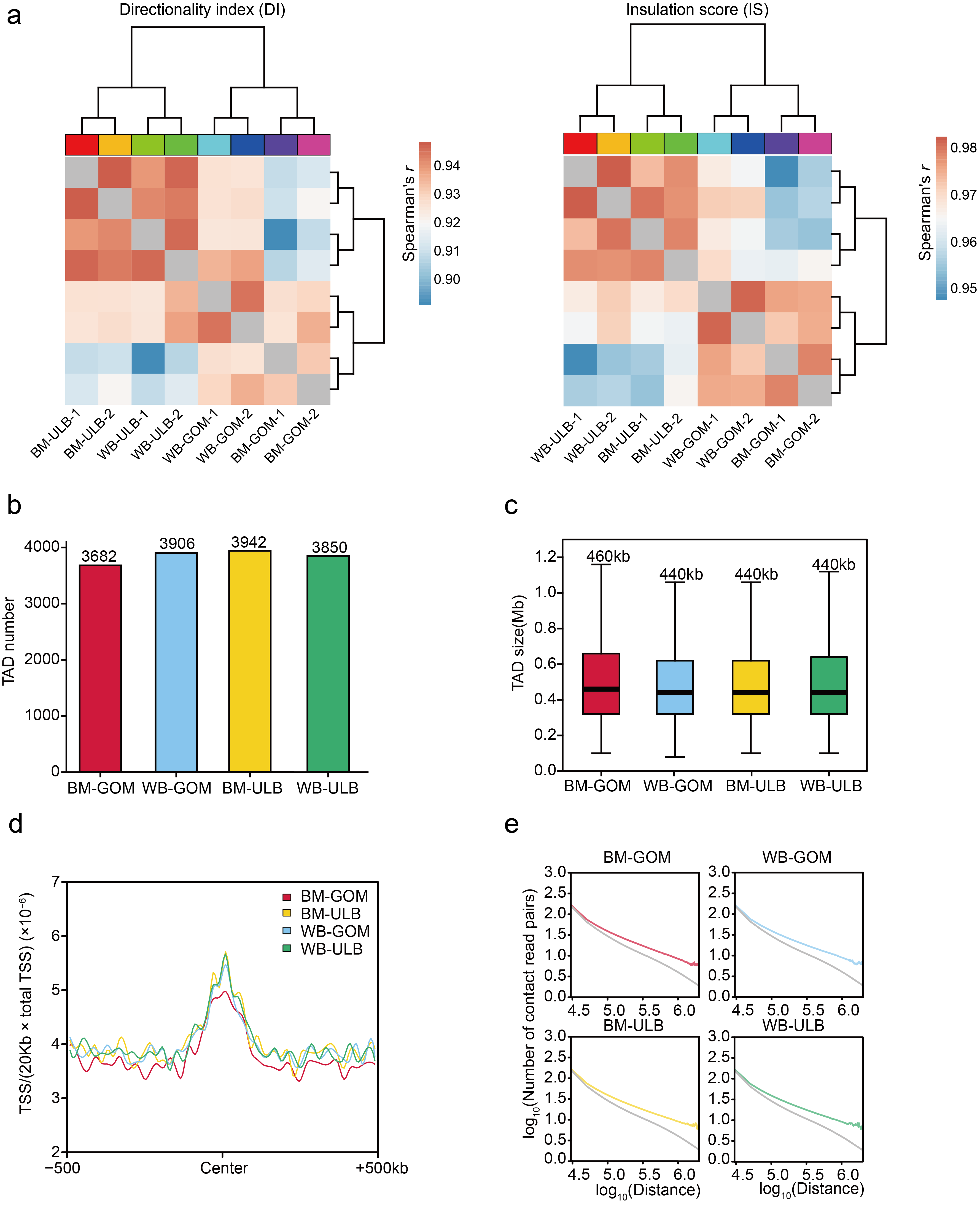


**Fig. S3 Characterization of TADs in wild boar and Bama pig ATs.**

(a) Spearman’s correlation heat map of the directionality index (DI) and insulation score (IS) between WB and BM ATs. (b) Bar plot showing the TAD number in each AT. (c) Distribution of TAD size in each AT. The horizontal line represents median TAD size, boxes indicate the 25th and 75th percentiles, and whiskers correspond to the 1.5× interquartile range. The median TAD size in each AT is shown above the boxplot. (d) The number of protein-coding genes surrounding boundary regions in each AT. (e) Log-log contact frequency that within or out of TAD as a function of the genomic distance (≤ 2 Mb) in each AT.


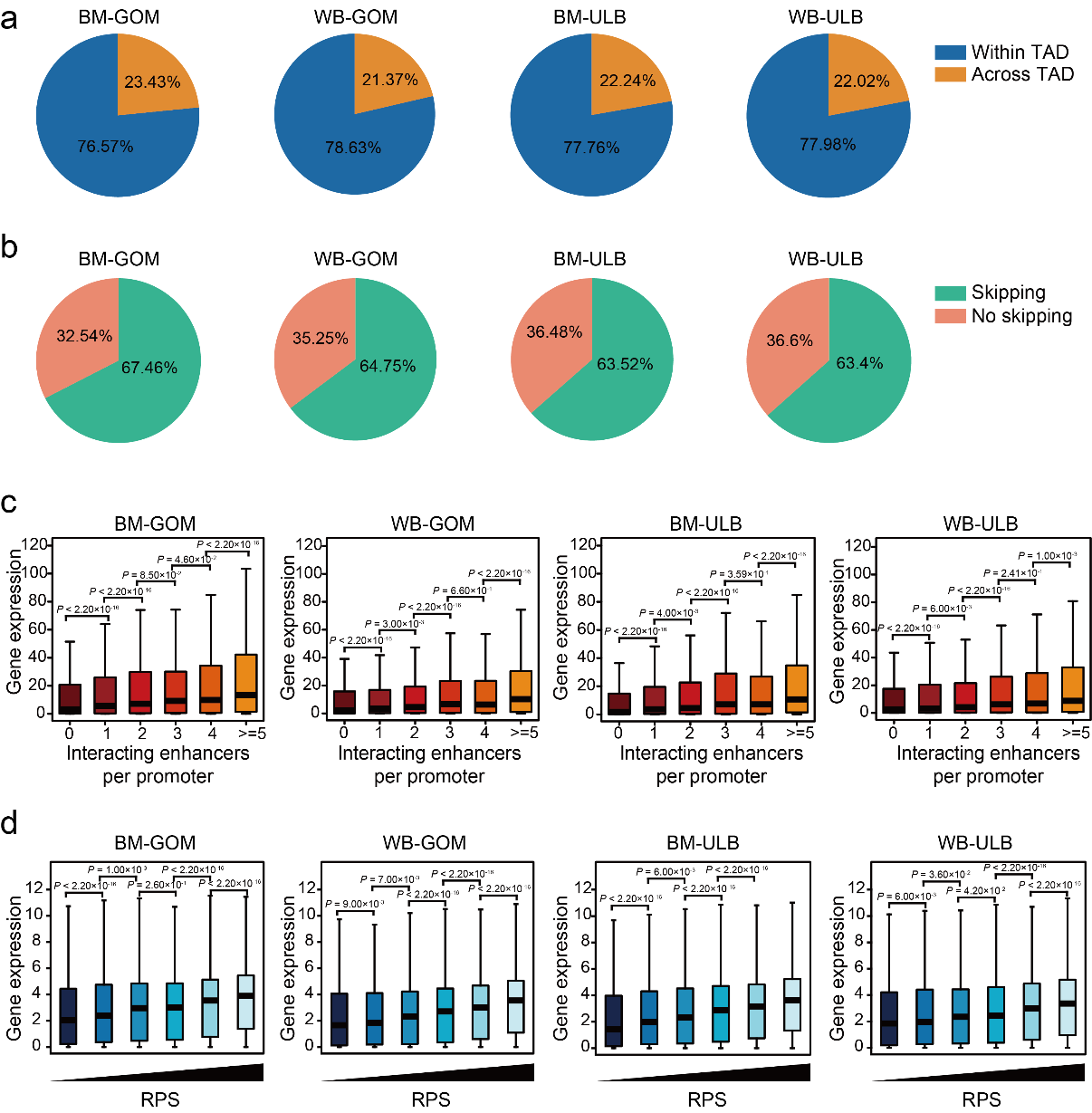


**Fig. S4 Characterization of PEIs.**

(a) Percentage of PEIs located within or across TADs. (b) Percentage of promoters interacting with the nearest putative enhancers (no-skipping) or those that skip at least one enhancer (skipping). (c) Expression level of genes with different interacting enhancer numbers in each AT. *P* values were calculated by Wilcoxon rank-sum test. (d) Expression of genes in different RPS categories in each AT. *P* values were calculated by Wilcoxon rank-sum test.

**
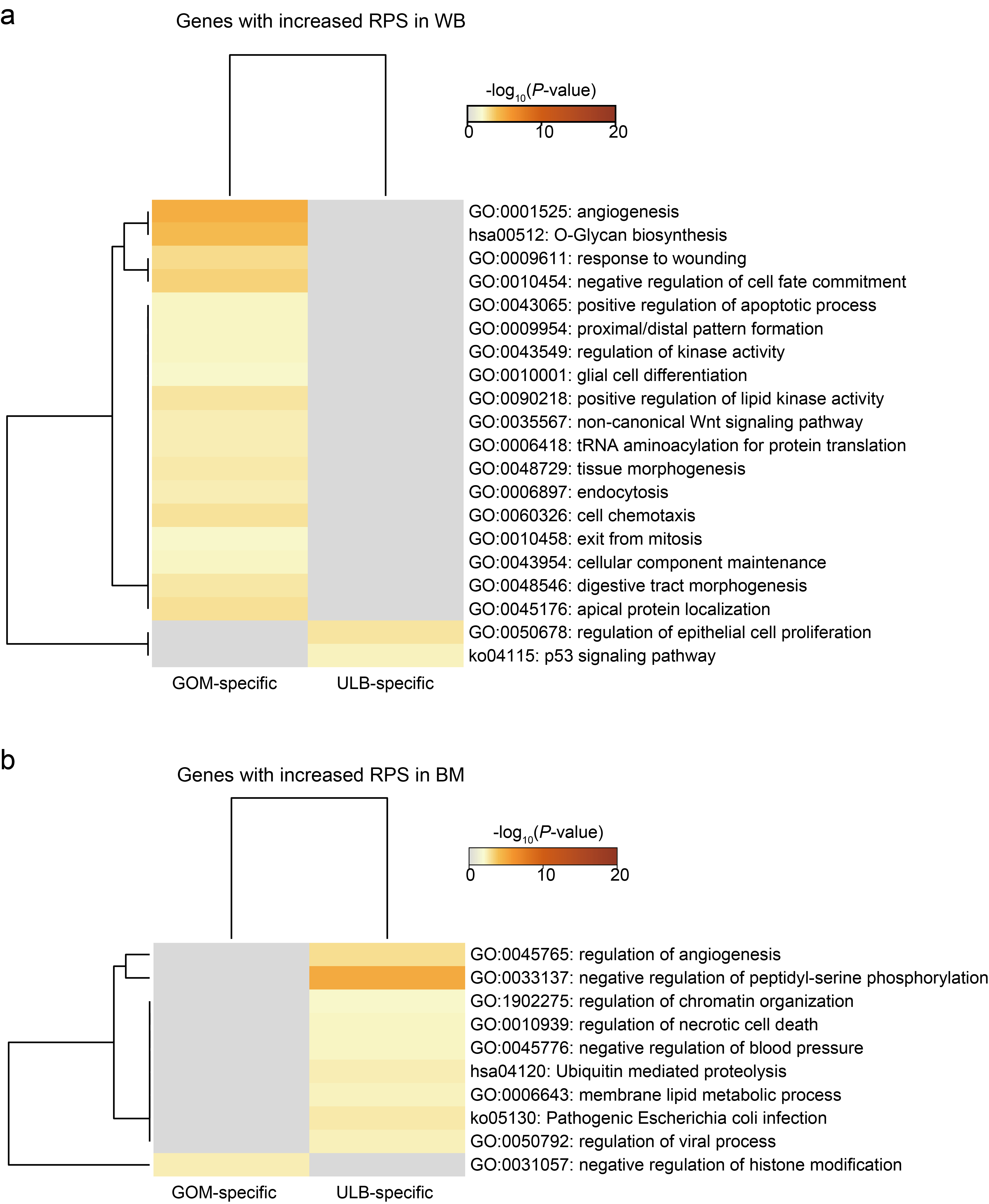
Fig. S5** Function of genes with ULB-specific or GOM-specific RPS changes cross wild boar and Bama pigs.
